# Supplementary material for: ProInfer: An interpretable protein inference tool leveraging on biological networks
Source: PLoS Comput Biol. 2023 Mar 17;19(3):e1010961. doi: 10.1371/journal.pcbi.1010961 (PMC10057851; doi:10.1371/journal.pcbi.1010961)
Supplement: Attachment — Submitted filename: response_letter.docx [file pcbi.1010961.s008.docx]

Dear Editor,

We would like to thank the reviewers and editorial for their valuable comments. We have revised our manuscript accordingly and the point-by-point responses are as follows.

**# Editor’s comments**

In particular I would suggest to focus on the issue of false positives raised by the reviewers and the performance comparison with other methods available. In addition is of paramount importance that your tools are accessible and usable without errors, therefore your github repository needs to be updated and kept up to date and flawless.

***Response:***

*We have added some tests to check the false positives raised by reviewer 2 and discussed the results in our Discussion at page 17. We already updated the source codes to fix the bugs and validated their correctness before renewing the GitHub repository. In addition, we have uploaded a stand-along version of ProInfer as suggested by reviewer1.*

When you are ready to resubmit, please upload the following:

[1] A letter containing a detailed list of your responses to the review comments and a description of the changes you have made in the manuscript. Please note while forming your response, if your article is accepted, you may have the opportunity to make the peer review history publicly available. The record will include editor decision letters (with reviews) and your responses to reviewer comments. If eligible, we will contact you to opt in or out.

***Response:***

*This is done.*

[2] Two versions of the revised manuscript: one with either highlights or tracked changes denoting where the text has been changed; the other a clean version (uploaded as the manuscript file).

***Response:***

*This is done. We have submitted two versions of revised manuscript with the names of “main_text_with_revision_highlights.docx” and “main_text_with_revision_clean.docx” (please see submissions).*

Figure Files:

While revising your submission, please upload your figure files to the Preflight Analysis and Conversion Engine (PACE) digital diagnostic tool, https://pacev2.apexcovantage.com. PACE helps ensure that figures meet PLOS requirements. To use PACE, you must first register as a user. Then, login and navigate to the UPLOAD tab, where you will find detailed instructions on how to use the tool. If you encounter any issues or have any questions when using PACE, please email us at [figures@plos.org](mailto:figures@plos.org).

***Response:***

*We already checked our figures with PACE, they all meet PLOS requirements*

Data Requirements:

Please note that, as a condition of publication, PLOS' data policy requires that you make available all data used to draw the conclusions outlined in your manuscript. Data must be deposited in an appropriate repository, included within the body of the manuscript, or uploaded as supporting information. This includes all numerical values that were used to generate graphs, histograms etc.. For an example in PLOS Biology see here: http://www.plosbiology.org/article/info%3Adoi%2F10.1371%2Fjournal.pbio.1001908#s5.

***Response:***

*we stored all the data for generating our figures in the file S1_Table.xlsx (see our Supporting Information)*

Reproducibility:

To enhance the reproducibility of your results, we recommend that you deposit your laboratory protocols in protocols.io, where a protocol can be assigned its own identifier (DOI) such that it can be cited independently in the future. Additionally, PLOS ONE offers an option to publish peer-reviewed clinical study protocols. Read more information on sharing protocols at <https://plos.org/protocols?utm_medium=editorial-email&utm_source=authorletters&utm_campaign=protocols>

***Response:***

*we don’t have laboratory protocols.*

**Comments of Review1**

**Reviewer #1**: The authors developed a protein inference tool named ProInfer. The proposed method makes use of prior knowledge from biological networks to improve the performance of protein assembly based on MS data. ProInfer showed superior performance compared to several other publically available tools. The tool was written in Python, and the source code is openly available. The work is of value, and ProInfer can be a valuable tool for proteomic data analysis.

To make the tool more accessible, especially to colleagues without computational background, I suggest, in addition to releasing the source code, providing a stand-alone version or a web-based application.

***Response to comments of Reviewer 1:***

*Thank you very much for your suggestion. Apart from our python codes, we have also released a stand-alone version application ProInfer_tk. One can download the ProInfer_tk.exe file from* [*https://drive.google.com/file/d/1GL3rXXADYKhjprHYwhGZ41_J-ThhsDOX/view?usp=sharing*](https://drive.google.com/file/d/1GL3rXXADYKhjprHYwhGZ41_J-ThhsDOX/view?usp=sharing) *(ProInfer.exe is too big to upload to GitHub) or run ProInfer_tk.py with python (see our GitHub repository:* [*https://github.com/PennHui2016/ProInfer*](https://github.com/PennHui2016/ProInfer)*).*

**Comments of Review2**

**Reviewer #2:** ProInfer is a computational analysis framework which infers the identification of proteins from ambiguous peptides identified in mass spectrometry data. The conceptual advance ProInfer provides is incorporating information from protein interaction networks such as CORUM (i.e. protein complexes) to improve the identification of proteins in a given sample. The authors’ compare their method against other protein inference algorithms on a benchmark of Human Protein Atlas validated/unvalidated proteins and show superior recall although limited relative precision. The authors’ also include an analysis of differentially expressed proteins and compare their results to other popular algorithms showing mostly positive performance.

Major:

***Q1:*** 1) Integration of protein complex information to improve protein identification may also introduce bias in the true positives and false positives proteins identified by the method. The authors’ alluded to some of the reasons in their discussion for true positive bias, in particular the incompleteness of biological networks. This however is not the only reason for bias, and I feel a full analysis of the false positives is necessary to give a complete picture.

One possible source of false positives is partners of proteins that participate in many complexes. It is unclear how these cases are handled in the ProInfer framework. This is a major concern due to the use of maximum probability values. Calmodulin (CALM1), for example, is found in 11 different Human Corum complexes many of them with non-overlapping subunits and unlikely to be expressed all together. If I understand the algorithm correctly, in a scenario where CALM1 is highly (or moderately) abundant in a sample, step 2 will assume all 11 CALM1 containing complexes are present regardless of if the complexes are actually formed, expressed, or seen previously in that cell type/condition. Further in Step 4, using the maximum will transfer the probability of the most confident protein (CALM1 in this case) to all other interaction partners in the 11 complexes regardless of if the individual subunits are expressed or not. This potentially is one source of false positives the method is producing.

***Response to Q1:***

*The complexes are signal enhancers to help transfer confidence scores among those likely co-expressed proteins, e.g., proteins in a common complex and* ***already have peptide evidence*** *(peptides from the protein and have been matched with LC-MS/MS spectra). Taking the CALM1 as an example (we assume it has a high confidence score s0 according to its peptide evidence, where s0 exceeds the threshold), we say it exists in 11 complexes {cp1, cp2, …,cp11}. There are another two protein P1 and P2 where P1 in cp1 and has a small confidence score s1 from its peptide evidence while P2 in cp2 with score s2. We say P1, P2 and CALM1 are possibly co-expressed in the complex c1 and c2, thus we update P1 and P2’s conference score as s0 (P1 and P2 are inferred as present). For other proteins that are also in these 11 complexes but having no peptide evidence, we ignore them. Thus, in practice, we will never infer whether a whole complex is existing, we only focus on those proteins in that complex and already have peptide evidence. In this way, the false positive rates are not inflated. In our future version of ProInfer, we may try to use an enrichment analysis or our previous weighted probability method to calculate a prior probability of whether a complex is existing, where those proteins in a complex without peptide evidence can also be inferred.*

*In addition, we tested three ways to transfer confidence scores such as using “minimum” (using smallest confidence score of proteins in a same complex), “maximum” and “mean” of its proteins’ confidence score. If minimum is used, then no protein’s confidence score will be updated (because a protein’s final confidence score is the larger one between its score from peptide evidence and from complex evidence). Comparing to method “mean”, we found that maximum always works much better when tested with our training data (Hela cell line, F1 is used as the performance metric, see our* ***S5 Table****) and “maximum” also runs faster than “mean”. Below is one example of the comparison testing on Hela DDA1 data.*

Comparison of mean and max for calculate complex confidence score

F1

| FDR(f)+psm_threshold(p) settings | mean | max |
| --- | --- | --- |
| f0.005p0.01 | 0.522925 | 0.522925 |
| f0.005p0.05 | 0.526198 | 0.526198 |
| f0.005p0.1 | 0.529459 | 0.529459 |
| f0.005p0.25 | 0.524812 | 0.531036 |
| f0.005p0.5 | 0.51795 | 0.5333 |
| f0.005p0.75 | 0.518565 | 0.529539 |
| f0.005p0.999 | 0.517334 | 0.546969 |
| f0.01p0.01 | 0.522925 | 0.522925 |
| f0.01p0.05 | 0.531811 | 0.531811 |
| f0.01p0.1 | 0.535253 | 0.535253 |
| f0.01p0.25 | 0.531456 | 0.535106 |
| f0.01p0.5 | 0.524342 | 0.536726 |
| f0.01p0.75 | 0.524271 | 0.542555 |
| f0.01p0.999 | 0.523629 | 0.553815 |
| f0.025p0.01 | 0.522925 | 0.522925 |
| f0.025p0.05 | 0.549878 | 0.549878 |
| f0.025p0.1 | 0.542162 | 0.542162 |
| f0.025p0.25 | 0.542569 | 0.545167 |
| f0.025p0.5 | 0.537688 | 0.54782 |
| f0.025p0.75 | 0.5375 | 0.550415 |
| f0.025p0.999 | 0.535951 | 0.560775 |
| f0.05p0.01 | 0.522925 | 0.522925 |
| f0.05p0.05 | 0.549878 | 0.549878 |
| f0.05p0.1 | 0.557624 | 0.557624 |
| f0.05p0.25 | 0.550199 | 0.551391 |
| f0.05p0.5 | 0.547547 | 0.553861 |
| f0.05p0.75 | 0.547052 | 0.556731 |
| f0.05p0.999 | 0.546942 | 0.571361 |

*We also discussed this comparison results in page 17 (highlighted in red in main_text_with_revision_highlights.docx). Below are the inserted discussions (newly inserted discussions are highlighted in red):*

Here, we simply assume the probability of a complex to be present equals to the **maximum** posterior probability of its proteins while the probability of a protein to be present from the complex side is measured as the **maximum** probability of all complexes containing it. In **S5 Table**, we tested setting a complex’s probability to be present as **mean** posterior probability of its proteins. However smaller F1 scores are always achieved especially when a higher psm threshold is configured. Though using mean helps reduce false positive rates (within 1%), more true positives are also dropped (~10%). Using maximum is currently an optimal selection, more advanced methods that help reduce false positives but keep true positives would be tried, e.g., calculate a prior probability of a complex to be present with enrichment test or our previous weighted probability method [34].

***Q2:*** An additional issue that is unclear is how the framework handles paralogous proteins. Paralogous proteins often share the same peptides and can participate as mutually exclusive partners in protein complexes. This method suggests if a partner in a shared complex is present, both paralogous proteins would be identified.

***Response to Q2:***

*The issue with paralogues is important but not one that is handled explicitly in ProInfer. We merely use protein complex information obtained from existing protein complex resources and make the naïve assumption that these complexes can be formed natively, without conflicting components that cannot co-exist simultaneously. Indeed, if the paralogous proteins do share the same peptides, we do not differentiate as our inference uses only peptide identification scores and complex composition information. The only way we may do so is if prior knowledge on paralogues and their tissue specificity can also be incorporated into ProInfer. This is interesting idea and is in line with ongoing work in our lab where we use gene expression information and ensemble learning methods to guide predictions on missing proteins, which are then verified using spectral information.*

*We have added the discussions of false positives caused by paralogous proteins in* ***Discussion****, in page 17 (highlighted in red in our main_text_with_revision_highlights.docx), below are detail added discussions:*

Thirdly, for paralogous proteins that share the same peptides and can participate as mutually exclusive partners in protein complexes [62], ProInfer may possibly infer them as either simultaneously present or absent. This is because ProInfer is dependent on prior knowledge based captured in the protein complex databases. It is possible to extend ProInfer by enriching the protein complex data with information on gene expression and paralogues. This may reduce potential false positives.

***Q3:*** Due to the above issues and possible other unknown biases, a more complete analysis of the false positives generated by the method is needed and a description of the biases introduced into the identified proteins due to the use of protein complexes as a source of integrated information.

***Response to Q3:***

*We added some new discussions in our* ***Discussion*** *section to describe the additional source of false positives. See red texts in page 17 (highlighted in main_text_with_revision_highlights.docx). Below are the added discussions:*

Here, we simply assume the probability of a complex to be present equals to the **maximum** posterior probability of its proteins while the probability of a protein to be present from the complex side is measured as the **maximum** probability of all complexes containing it. In **S5 Table**, we tested setting a complex’s probability to be present as the **mean** posterior probability of its proteins. However smaller F1 scores are always achieved especially when a higher psm threshold is configured. Though using mean helps reduce false positive rates (within 1%), much more true positives are also dropped (~10%). Using maximum is currently an optimal selection, more advanced methods that help reduce false positives but keep true positives could be tried, e.g., calculate a prior probability of a complex to be present with enrichment test or our previous weighted probability method [34].

Thirdly, for paralogous proteins that share the same peptides and can participate as mutually exclusive partners in protein complexes [62], ProInfer may possibly infer them as either simultaneously present or absent. This is because ProInfer is dependent on prior knowledge captured in the protein complex databases. It is possible to extend ProInfer by enriching protein complex data with information on gene expression and also paralogs. This may reduce potential false positives.

62. Ori, A., Iskar, M., Buczak, K., Kastritis, P., Parca, L., Andrés-Pons, A., ... & Beck, M. (2016). Spatiotemporal variation of mammalian protein complex stoichiometries. Genome biology, 17(1), 1-15.

***Q4:*** 2) It is unclear how the decoy protein complexes are utilized in the method. It is mentioned but not fully described.

***Response to Q4:***

*Decoy proteins are added for controlling FDR in protein inference. We used protein complex information to enhance protein inference, and only target proteins which reside in protein complexes. We generate decoy complexes and update the decoy proteins’ confidence scores in the similar way as updating target proteins’ confidence scores to avoid introducing bias in FDR measurement when incorporating protein complex information. So,* ***the utilization of decoy complexes starts from checking whether decoy proteins are co-expressed in one or more decoy complexes and then we update those possibly co-expressed decoy proteins’ confidence scores. Finally, a decoy protein’s confidence score is the maximum value between its scores from peptides and from decoy complexes.*** *We added descriptions about the use of decoy proteins in page 9 (highlighted in red in our main_text_with_revision_highlights.docx). Below are the revised descriptions (inserted sentences are in red):*

For decoy proteins, PP derived from corresponding decoy complexes will be used (similarly, calculated by max(original PP, complexes’ PPs)). A decoy complex is constructed by replacing target proteins in its twin true complex with decoy ones. The introducing of decoy complexes is to make the inference of both target proteins and decoy proteins in a similar way to avoid bias in estimating FDR.

***Q5:*** 3) The Human Protein Atlas is used as a benchmark. It is unclear which dataset within the HPA was used. A link to the downloads page would aid in replicability. Further, the description of “validated” vs “non-validated” is vague. What do the authors’ mean by “different tools”? Is it only antibody based methods? Specifics are required for a full evaluation of the method. Publishing the full benchmark in the supplemental along with ProInfer’s identified proteins is necessary as well.

***Response to Q5:***

*We used the cell line transcriptome data that shows both protein-coding gene RNA expression profiles and whether there are protein level evidence of proteins’ expression, the Hela related data were downloaded via link* [*https://www.proteinatlas.org/search/NOT+celline_category_rna%3A*](https://www.proteinatlas.org/search/NOT+celline_category_rna%3AHeLa%3BNot+detected) *HeLa%3BNot+detected* *and THP1 data were downloaded via link* [*https://www.proteinatlas.org/sea*](https://www.proteinatlas.org/search/NOT+celline_category_rna%3ATHP-1%3BNot+detected)

*rch/NOT+celline_category_rna%3ATHP-1%3BNot+detected*. *Here, “validated” and “non-validated” are labeled by protein inference tools such as our ProInfer, where if a protein is existing in Hela cell line from HPA and it was inferred to be existing by ProInfer for a given FDR threshold, then we say our ProInfer’s inference of this protein’s existing is validated, otherwise non-validated. The “different tools” refer to those completing protein inference tools such as our ProInfer, EPIFANY etc. We have attached the protein lists for protein inference validation in our* ***S4 Table****. The validation results with the alternative validation sets generated by filtering out protein in HPA but without protein level evidence are presented in our* ***S7 Table*** *(see below additional revisions 36). We also revised the description of HPA and protein inference validation as follows (red texts are revised, we also highlighted them in red in the main_text_with_revision_highlights.docx in page 10):*

Positive proteins for Hela cell line were downloaded (data were downloaded from https://www.proteinatlas.org/search/NOT+celline_category_rna%3AHeLa%3BNot+detected). Proteins having UniProt ids [25] were retained. For Hela cell line, there are 11806 validated proteins (See detail protein list in **S4 Table**). We label the proteins predicted by different protein inference tools, e.g., our ProInfer, EPIFANY, etc., and validated by the Human Protein Atlas as “Validated” (validated proteins are true positives), otherwise they are considered “Non-validated” (false positives). We calculate several metrics for evaluating competing tools and optimizing their hyperparameters including inferred protein numbers, validated protein numbers, recall, precision and F1 score. Recall, precision and F1 score are given by:

recall = TP/(TP+FN)	(16)

precision = TP/(TP+FP)	(17)

F1 = 2 * recall * precision/(recall + precision)	(18)

Where, a TP (true positive) refers to a protein reported by an inference tool, e.g., our ProInfer and validated by HPA, FN (false negative) means a protein in HPA but has not been reported by a given protein inference tool, and FP is the protein not in HPA but has been reported.

*In page 12 we also added the download link of HPA data for cell line THP1, below text in red are revised part (also highlighted in page 12 of main_text_with_revision_highlights.docx):*

Positive proteins were obtained from HPA (11584 proteins, see **S4 Table**, data can be downloaded via <https://www.proteinatlas.org/search/NOT+celline_category_rna%3ATHP-1%3BNot+detected>). For each tool, optimal hyperparameters were determined as described above. The average performance across the 3 M0 THP1 replicates were used. In addition, an alternative positive protein set generated by filtering out proteins in HPA and without protein level evidence (with 11483 proteins) were also tested, minor performance differences were obtained, see **S7 Table** for more details.

***Q6:*** 4) The text states “… literature proofs were searched to prove they really associate with lung cancer.” It is unclear what the criterion is for inclusion here. Many studies have extremely weak associations between genes and diseases and rigorous review of the literature is required to ensure strong evidence of associations. The authors should detail their method and include a supplemental table for the reader to evaluate.

***Response to Q6:***

*We searched the literatures with key words of a given protein (or its gene) and lung cancer, we read the paper to find out whether there is a conclusion that the protein is associated with the cancer, if yes, we regarded it as evidence of supporting. We attach the evidence in* ***S3 Table*** *(where the exact sentences obtained in-text are added). We added the description of our literature validation process in page 14 (highlighted in red in main_text_with_revision_highlights.docx ). Below are the inserted descriptions (red texts are newly inserted):*

These unique DEPs may be valuable, e.g., novel biomarkers or drug targets. We drew an additional Venn diagram (**Figure 3D** inset) to check the validation status of these unique DEPs, where literature proofs were searched to prove they really associate with lung cancer. We searched literature by key words of given protein and lung cancer at first. Then we read the literature to check whether they reported any correlations between the protein and lung cancer.

***Q7:*** 5) A fresh clone of the github repository does not produce executable code. I received an error when trying the example command: python ProInfer.py ./DDA1.tsv

NameError: name 'argparse' is not defined

When I added ‘import argparse’ to the file, I received a different error:

ProInfer.py: error: unrecognized arguments: ./DDA1.tsv

***Response to Q7:***

*We have fixed the errors in our source codes (ProInfer.py and OpenMS_ProInfer.py) in GitHub repository (*[*https://github.com/PennHui2016/ProInfer*](https://github.com/PennHui2016/ProInfer)*). We also provide a stand-alone version of ProInfer (ProInfer_tk) based on the “Tkinter” python package, users can run “python ProInfer_tk.py” directly or run ProInfer_tk.exe with a Windows OS and then fill the required parameters to use our tool. We hope this will facilitate more researchers to use this tool.*

Minor:

***Q8:*** Some of the axes in the figures are not labeled properly. For example in Figure 3A-C, the x-axis requires a label.

***Response to Q8:***

*We already added labels of the figures in page 15 of our manuscript (we highlight the caption of this figure in our main_text_with_revision_highlights.docx) below is the new figure:*

**Figure 3** **Evaluation of methods on differential expression analysis**. A, B & C show the numbers of differentially expressed proteins (DEPs) and non-differentially expressed proteins (Non-DEPs) found by different protein inference tool-based, FragPipe and maxquant-based workflows from lung cancer, RAW264.7 and THP1 cell line data. D shows overlapping of DEPs reported by different workflows from lung cancer data and the validation status of those uniquely found DEPs. E gives the top10 example validation proofs of DEPs uniquely found by ProInfer.

**Comments of Review3**

**Reviewer #3:** In this article, the authors present ProInfer, a method that performs protein identification by using an inference method harnessing protein complex information.

The methods are interesting, and well explained, and the results appear convincing. I have a few comments that I believe should be addressed:

***Q1:*** - the independence assumption in equation 6 seems entirely unjustified, especially if overlapping peptides are considered. Can the authors test that assumption? This directly relates to the next point, since this formula is used for the computation of the FDR:

***Response to Q1:***

*We agree the approach is not perfect, but our purpose is also not to reinvent this portion by proposing a new evidence aggregation method.*

*The approach we used for combining peptide information to determine the confidence score of a protein is a classical method to integrate multiple peptide evidence in protein assembly. See an example literature of “Nesvizhskii et al. A Statistical Model for Identifying Proteins by Tandem Mass Spectrometry. Analytical chemistry, 75(17), 4646-4658”. Thus, we also calculate our accumulate PEP scores in similar way.*

***Q2:*** - The flat precision curve for ProInfer in figure 2B is puzzling: the precision should go down as the FDR increase. How do the author explain this behaviour?

***Response to Q2:***

*In Figure 2B we show the average (we have three replicates of M0 data, see our Table 1) precision scores obtained by different protein inference tools under four protein reporting FDRs. Our precision for FDR 0.005, 0.01, 0.025 and 0.05 are 0.935, 0.935, 0.936 and 0.935 respectively. There is an increase when FDR increases from 0.01 to 0.025. This is because we inferred more validated proteins than non-validated ones. See Figure 2A, when FDR increases, non-validated protein numbers have nearly no change, while validated protein numbers increase.*

***Q3:*** - It seems to me that the "reliable" complexes are implicitely given a prior of 1, and the same weight. But different complexes have widely varying concentrations, and confidence. Are they all given the same prior likelihood ?

***Response to Q3:***

*Our objective of using protein complexes is to form a co-expression network that helps transfer protein existing confidence scores to those proteins having peptide evidence and are possibly co-expressing. So, we are not too concerned whether the whole complex is formed or not, with stoichiometric precision. As our goal here is protein inference, which is to determine whether a protein is present or not, the complex or protein’s concentration is not considered explicitly.*

*With that being said, the stoichiometry and abundance can be useful for understanding the factor for initial non-observation. It may also be used for determining correlations amongst members of a protein complex, which in turn, can be used to determine the likelihood of a protein being present, given observation of its strongly/less-correlated same complex members. However, such information is not present in the protein complex database we used.*

***Q4:*** - how useful is proinfer when pre-existing identifications of proteins are weaker? for example with less identified proteins in the reference? and how does it affect ProInfer's precision and recall? It seems hard to tell whether ProInfer is efficiently using weak peptide identifications, or using noisy peptide spectra to assign proteins from the network. (remove some targets)

***Response to Q4:***

*We use the DDA1 data to test how our ProInfer performs when pre-existing identifications of proteins are weaker. We regarded target proteins identified with only peptide evidence as pre-existing identifications prior to integration with protein complex. We remove 1%~50% of these target proteins where they are used to simulate weaker identifications. Following two figures show the changes of precisions and recalls due to the weaker identifications. We can see at FDR 0.005, when 50% of the target proteins are weaker identifications, precision even increases from 0.9609 to 0.9610, at the same time recall drops from 0.3823 to 0.3204. (See our* ***S6 Table.****) In comparison, at FDR 0.05, precision decreases 0.0034 while recall decreases 0.0700 when 50% of the pre-existing proteins are removed. Thus, our ProInfer are robust to weaker pre-existing identifications.*

Removal of target proteins identified from peptide evidence only

Precision

| FDR | original | remove_1% | remove_5% | remove_10% | remove_20% | remove_50% |
| --- | --- | --- | --- | --- | --- | --- |
| 0.005 | 0.960906 | 0.96115 | 0.960951 | 0.961077 | 0.960462 | 0.960999 |
| 0.01 | 0.960949 | 0.960995 | 0.960704 | 0.960535 | 0.960869 | 0.961196 |
| 0.025 | 0.959062 | 0.959677 | 0.954713 | 0.953994 | 0.953836 | 0.953271 |
| 0.05 | 0.949447 | 0.949405 | 0.948728 | 0.948432 | 0.948332 | 0.946094 |

Removal of target proteins identified from peptide evidence only

Recall

| FDR | original | remove_1% | remove_5% | remove_10% | remove_20% | remove_50% |
| --- | --- | --- | --- | --- | --- | --- |
| 0.005 | 0.382288 | 0.380588 | 0.374384 | 0.367245 | 0.353051 | 0.320415 |
| 0.01 | 0.389002 | 0.387387 | 0.380248 | 0.372344 | 0.36104 | 0.330529 |
| 0.025 | 0.396226 | 0.394442 | 0.395971 | 0.387727 | 0.374044 | 0.338093 |
| 0.05 | 0.408635 | 0.40668 | 0.399456 | 0.390787 | 0.374384 | 0.338603 |

***Q5:*** - it would be useful to see a comparison between proInfer and some of the other tools as a function of psm threshold: does proInfer do better than other tools at smaller psm thresholds?

***Response to Q5:***

*We regarded the psm threshold as a hyperparameter for choosing a set of PSMs for further protein inference. In fact, during our hyperparameter choosing stage with the Hela cell line data, we already tested how different tools work under different psm thresholds. Our ProInfer, Percolator and PIA prefer higher psm thresholds while EPIFANY and Fido work better with a smaller psm threshold (see our* ***S2 Table*** *supp.tab1~* *supp.tab5).*

***Q6:*** - It would be nice to have in supplementary some peptide spectra, to illustrate what type of evidence that proInfer uses to draw its inference, that would otherwise be ignored.

***Response to Q6:***

*In fact, protein inferences are not based directly on spectra themselves. Most of existing protein inference tools including our ProInfer just use peptide identification confidence scores (e.g., posterior error probability (PEP) ) and peptide-protein connections. Spectra are used in database search process, so they are useless to protein inference stage.*

***Additional revisions***

*We also corrected some grammar errors in our manuscript. We highlighted these modifications in file main_text_with_revision_highlights.docx in red. Following are tracks of our modifications, we underline those original contents that have been modified in revised version and color corresponding revised contents in red (page numbers are counted in main_text_with_revision_highlights.docx):*

1.) *We changed the order of author affiliations,*

*Previous:*

Hui Peng ^1,3^^, Limsoon Wong ^2*^, Wilson Wen Bin Goh^1,3,4*^

1. Lee Kong Chian School of Medicine, Nanyang Technological University, Singapore
2. Department of Computer Science, National University of Singapore, Singapore
3. School of Biological Sciences, Nanyang Technological University, Singapore
4. Center for Biomedical Informatics, Nanyang Technological University, Singapore

*Revised:*

Hui Peng ^1,2^^, Limsoon Wong ^3*^, Wilson Wen Bin Goh^1,2,4*^

1. Lee Kong Chian School of Medicine, Nanyang Technological University, Singapore
2. School of Biological Sciences, Nanyang Technological University, Singapore
3. Department of Computer Science, National University of Singapore, Singapore
4. Center for Biomedical Informatics, Nanyang Technological University, Singapore
2.) *In* ***Abstract*** *at page 3,*

*Previous:*

We present ProInfer (Protein Inference), a novel protein assembly method that takes advantage of information biological networks.

*Revised:*

We present ProInfer (Protein Inference), a novel protein assembly method that takes advantage of information in biological networks.

3.) *In* ***Abstract*** *at page 3,*

*Previous:*

ProInfer assists recovery of proteins supported only by ambiguous peptides (a peptide which maps to more than one candidate protein) and enhances the statistical confidences for proteins supported by both unique and ambiguous peptides.

*Revised:*

ProInfer assists recovery of proteins supported only by ambiguous peptides (a peptide which maps to more than one candidate protein) and enhances the statistical confidence for proteins supported by both unique and ambiguous peptides.

4.) *In* ***Abstract*** *at page 3,*

*Previous:*

Evaluated across THP1 cell line, lung cancer and RAW267.4 datasets, ProInfer always infers the most validatable proteins compared against mainstream protein inference tools Fido, EPIFANY and PIA.

*Revised:*

Evaluated across THP1 cell line, lung cancer and RAW267.4 datasets, ProInfer always infers the most numbers of validated proteins, in comparison to mainstream protein inference tools Fido, EPIFANY and PIA.

5.) *In* ***Author summary*** *at page 3,*

*Previous:*

However, this procedure suffers from coverage issues due to high statistical stringencies and noise.

*Revised:*

However, this procedure suffers from coverage issues due to high statistical stringency requirement and noise.

6.) *In* ***Author summary*** *at page 3,*

*Previous:*

Comparing with existing tools, ProInfer achieves the highest recall and F1 scores in protein inference but also identifies novel differentially expressed proteins not reported by any other tool.

*Revised:*

Compared against existing tools, ProInfer achieves the highest recall and F1 score in protein inference and also identifies novel differentially expressed proteins not reported by any other tool.

7.) *In the second paragraph of page 4,*

*Previous:*

For experimental/biological, example challenges include incomplete proteome coverage due to high dynamic range of protein abundances, limitations in digestion and protein separability under experimental running conditions, and detector sensitivity and resolution [13]. For analytical, the algorithm design and assumption validity, parameter values, and sequence library completeness are example challenges.

*Revised:*

Example experimental/biological challenges include incomplete proteome coverage due to high dynamic range of protein abundances, limitations in digestion and protein separability under experimental running conditions, and detector sensitivity and resolution [13], whereas the algorithm design and assumption validity, parameter values, and sequence library completeness are example analytical challenges.

8.) *In the second paragraph of* *page 4,*

*Previous:*

Peptide ambiguity problem is solved by either discarding ambiguous peptides (peptides which map to > 1 protein) entirely, e.g., Percolator [9] or conducting network analysis on peptide-protein bipartite networks, e.g., EPIFANY [13]. Those proteins sharing the same constituent peptides may be reported as protein groups [12] in which case, we can be assured at least 1 member in the protein group exists.

*Revised:*

Peptide ambiguity problem is solved by either discarding ambiguous peptides (peptides which map to > 1 protein), e.g., Percolator [9]; or conducting network analysis on peptide-protein bipartite networks, e.g., EPIFANY [13]. Those proteins sharing the same constituent peptides may be reported as protein groups [12] in which case, we can be assured that at least 1 member in the protein group exists.

9.) *In the first paragraph of page 5,*

*Previous:*

To overcome these information barriers, we believe incorporating prior knowledge via data integration, e.g., drawing from independent data sources such as biological networks is essential.

*Revised:*

To overcome these information barriers, we believe incorporating prior knowledge via data integration, e.g., drawing from independent data sources such as biological networks, is essential.

10.) *At the end of the first paragraph in page 5,*

*Previous:*

Additionally, ProInfer borrows similar principles from our missing protein prediction method PROTREC [34] that leverages on the phenomenon that proteins forming a stable protein complex (or constituting part of a tightly clustered network module) are more likely to be co-expressed [35], we incorporated protein complex information to rescue proteins with weak signals themselves but have neighbors with strong evidence. Tested across several datasets, we demonstrate ProInfer achieves excellent performance in both protein inference and downstream quantitative analysis.

*Revised:*

Additionally, ProInfer borrows similar principles from our missing protein prediction method PROTREC [34] that leverages on the phenomenon that proteins forming a stable protein complex (or constituting part of a tightly clustered network module) are more likely to be co-expressed [35]. Specifically, it incorporates protein complex information to rescue proteins with weak signals themselves but have neighbors with strong evidence. ProInfer achieves excellent performance in both protein inference and downstream quantitative analysis.

11.) *In the first paragraph of* ***Materials and Methods*** *in page 5,*

*Previous:*

And thus, the protein’s existence adds evidence supporting the presence of the complex in a tissue (i.e., complex inference process based on observed constituent proteins). Alternatively, an arrow from a protein complex to a protein propagates the existing probability of this complex to its constituent protein, helping re-evaluate our confidence in the protein’s existence in a tissue (i.e., protein rescoring).

*Revised:*

And thus, the protein’s existence adds evidence supporting the presence of the complex in a tissue (i.e., protein complex inference process based on observed constituent proteins). Alternatively, an arrow from a protein complex to a protein propagates the existence probability of this complex to its constituent proteins, helping to re-evaluate our confidence in the protein’s existence in a tissue (i.e., protein rescoring).

12.) *In the last paragraph of page 5,*

*Previous:*

The false-reporting probability of using Q to support reporting $P_{1}$ is the chance that *Q* is an incorrect identification (with PEP score of ${pep}_{Q}$) plus the chance that *Q* is not an incorrect but does not come from $P_{1}$ (i.e., it is from one of $P_{2}, \ldots, P_{N}$).

*Revised:*

The false-reporting probability of using Q to support reporting $P_{1}$ is the chance that *Q* is an incorrect identification (with PEP score of ${pep}_{Q}$) plus the chance that *Q* is not incorrect but does not come from $P_{1}$ (i.e., it is from one of $P_{2}, \ldots, P_{N}$).

13.) *In the middle of page 6,*

*Previous:*

If each peptide $Q_{j}$supports a protein $P_{1}$ uniquely, this peptide is considered “unique”.

*Revised:*

If a peptide $Q_{j}$supports a protein $P_{1}$ uniquely, this peptide is considered “unique”.

14.) *In the section “Complex inference and protein rescoring with protein-complex network” in page 7,*

*Previous title:*

Complex inference and protein rescoring with protein-complex network

*Revised title:*

Protein complex inference and protein rescoring with protein-complex network

15.) *In section “Complex inference and protein rescoring with protein-complex network” in page 7,*

*Previous:*

In a typical experiment setting, due to limitations in instrument sensitivity, protein abundance and protein sequence uniqueness, some proteins are only supported by weak signals e.g., few supporting peptides and/or low confidence peptides, and thus, are difficult to observe.

*Revised:*

In a typical experiment setting, due to limitations in instrument sensitivity, protein abundance and protein sequence uniqueness, some proteins are only supported by weak signals (e.g., few supporting peptides and/or low confidence peptides), and thus, are difficult to observe.

16.) *In section “Complex inference and protein rescoring with protein-complex network” in page 7,*

*Previous:*

We hypothesize protein complexes can also be useful for improving protein inference from peptide information. To test this idea, we use protein complex information in ProInfer.

Assuming ProInfer (refers to the part described in above section) outputs L candidate proteins and their accPEP scores and q-values denoted by:

*Revised:*

We hypothesize that protein complexes can also be useful for improving protein inference from peptide information. To test this idea, we use protein complex information in ProInfer.

Suppose ProInfer (refers to the part described in above section) outputs L candidate proteins and their accPEP scores and q-values denoted by:

17.) *In last paragraph of page 7,*

*Previous:*

We collected *C* reliable protein complexes and generate *C* decoy protein complexes by replacing

*Revised:*

We collected *C* reliable protein complexes and generated *C* decoy protein complexes by replacing

18.) *The sentence below formula (11) in page 8,*

*Previous:*

where $c_{j}$ is the *j*th known complexes, and it contains *X* known proteins denoted by ${cP}^{j}=\{{cP}_{1}^{j}, {cP}_{2}^{j},\ldots,{cP}_{X}^{j}\}$.

*Revised:*

where $c_{j}$ is the *j*th known protein complexes, and it contains *X* constituent proteins denoted by ${cP}^{j}=\{{cP}_{1}^{j}, {cP}_{2}^{j},\ldots,{cP}_{X}^{j}\}$.

19.) ***Step3*** *in page 8,*

*Previous:*

**Step3.** Calculate the probability of protein $P_{i}$ in $Pros$ being present in the sample according to the complex information.

*Revised:*

**Step3.** Calculate the probability of protein $P_{i}$ in $Pros$ being present in the sample according to protein complex information.

20.) ***Step3*** *in page 8,*

*Previous:*

If no complex contains $P_{i},$ then $p_{cpx\left( P_{i} \right)}=0$.

*Revised:*

If no protein complex contains $P_{i},$ then $p\_cpx\left( P_{i} \right)=0.$

21.) ***In the first paragraph of p****age 9,*

*Previous:*

For decoy proteins, PP derived from corresponding decoy complexes will be used. A decoy complex is constructed by replacing target proteins in its twin true complex with decoy ones. This propagative procedure is iterated until no additional target proteins are reported under a given FDR.

*Revised:*

For decoy proteins, PP derived from corresponding decoy complexes will be used (similarly, calculated by max(original PP, complexes’ PPs)). A decoy complex is constructed by replacing target proteins in its twin true complex with decoy ones. The introduction of decoy complexes is to make the inference of both target proteins and decoy proteins in a similar way to avoid bias in estimating FDR. This propagation procedure is iterated until no additional target proteins are reported under a given FDR.

22.) *In the third paragraph of page 9,*

*Previous:*

For fair comparison, we optimized their hyperparameters with the same Hela cell line dataset initially. Hela cell line, derived from cervical cancer cells, is the oldest and most used human cell line [36], is well-documented and widely applied in biochemical, biological, and medical experiments [37].

*Revised:*

For a fair comparison, we optimized their hyperparameters with the same Hela cell line dataset initially. The Hela cell line, derived from cervical cancer cells, is the oldest and most used human cell line [36]; it is well-documented and widely applied in biochemical, biological, and medical experiments [37].

23.) *In the first paragraph of page 10,*

*Previous:*

PSM filtering thresholds (PSMs with PEP scores bigger than the threshold are dropped) were ranged among [0.01, 0.05, 0.1, 0.25, 0.5, 0.75, 0.999], while other tool-specific hyperparameters, all possible values are tested.

*Revised:*

PSM filtering thresholds (PSMs with PEP scores bigger than the threshold are dropped) were ranged among [0.01, 0.05, 0.1, 0.25, 0.5, 0.75, 0.999], while for other tool-specific hyperparameters, all possible values are tested.

24.) *In the last paragraph of page 11,*

*Previous:*

We identified the optimal running conditions (or settings) for each tool (ProInfer, EPIFANY, Fido, Percolator and PIA.) given data of a particular nature.

*Revised:*

We identified the optimal running conditions (or settings) for each tool (ProInfer, EPIFANY, Fido, Percolator and PIA) given data of a particular nature.

25.) *In the third paragraph of page 12,*

*Previous:*

We tuned hyperparameters for EPIFANY, Fido, Percolator and PIA accordingly. EPIFANY works best when set PSM PEP<=0.05 and parameter greedy_group_resolution to be “remove_associations_only” and without regularize. For Fido, its optimal hyperparameters are: PSM PEP<=0.1 and greedy_group_resolution setting as “true”. Like ProInfer, Percolator also works best with PSM PEP<=0.999. For PIA, inference method of Spectrum Extractor, multiplicative scoring method and PSM PEP score are chosen, and PEP<=0.999 is used. More details are found in **Supplementary file 1.**

*Revised:*

We tuned hyperparameters for EPIFANY, Fido, Percolator and PIA accordingly. EPIFANY works best when setting PSM PEP<=0.05 and parameter greedy_group_resolution to be “remove_associations_only” and without regularize. For Fido, its optimal hyperparameters are: PSM PEP<=0.1 and greedy_group_resolution setting as “true”. Like ProInfer, Percolator also works best with PSM PEP<=0.999. For PIA, inference method of Spectrum Extractor, multiplicative scoring method and PSM PEP score are chosen, and PEP<=0.999 is used. More details are found in **S2 Table.**

26.) *In the last paragraph of page 12 and the first paragraph in page 13,*

*Previous:*

For instance, given FDR 0.01, ProInfer reports 5390 proteins in total, of which, 5041 is validatable. Compared against Percolator, 640 (**Figure 2A**, 5041-4401) more validated proteins were reported with just 230 (**Figure 2A**, 349-119) more non-validated ones, achieving a 1:2.78 ratio of non-validated: validated gain (230:640).

*Revised:*

For instance, given FDR 0.01, ProInfer reports 5390 proteins in total, of which, 5041 are validated proteins. Compared against Percolator, 640 (**Figure 2A**, 5041-4401) more validated proteins were reported with just 230 (**Figure 2A**, 349-119) more non-validated ones, achieving a 1:2.78 ratio of non-validated:validated gain (230:640).

27.) *In the first paragraph of page 13,*

*Previous:*

In addition, from **Figure 2A** we can see, even though with looser FDR thresholds, e.g., 0.025 and 0.05, ProInfer reports more validatable proteins without incurring great changes to the presence of non-validatable proteins (about 10 more non-validated comparing to FDR 0.005 or 0.01).

*Revised:*

In addition, from **Figure 2A**, even with looser FDR thresholds, e.g., 0.025 and 0.05, ProInfer reports more validated proteins without incurring great changes to the presence of non-validated proteins (about 10 more non-validated comparing to FDR 0.005 or 0.01).

28.) *In Figure 2E, we wrongly counted a blank as a protein for PIA’s inference result, the correct number of proteins inferred by PIA is 4813 (previous is 4814) and the number of proteins uniquely inferred by PIA is 166 (previous is 167). For the validation by HPA, 96 out of these 166 proteins are also detectable in HPA and remaining 70 (previous is 71) are non-detectable.*

*Previous:*

*Revised:*

29.) *In the first paragraph of page 14,*

*Previous:*

PIA uniquely reported just 167 proteins, with 57.5% (96 out of 167) validated.

*Revised:*

PIA uniquely reported just 166 proteins, with 57.8% (96 out of 166) validated.

30.) *In the last paragraph of page 14,*

*Previous:*

71 DEPs were reported by all 7 workflows, composed of 54% of all DEPs found by EPIFANY- based workflow (highest) and 32% of ProInfer-based workflow (lowest).

*Revised:*

71 DEPs were reported by all 7 workflows, comprising 54% of all DEPs found by EPIFANY- based workflow (highest) and 32% of ProInfer-based workflow (lowest).

31.) *In the first paragraph of page 15,*

*Previous:*

More details about the literature proofs for these uniquely reported DEPs can be found in **Supplementary file 2.**

*Revised:*

More details about the literature proofs for these uniquely reported DEPs can be found in **S3 Table.**

32.) *In the second paragraph of page 16,*

*Previous:*

In our evaluations, this strategy has proven effective in identifying more validatable proteins than existing tools.

*Revised:*

In our evaluations, this strategy has proven effective in identifying more validated proteins than existing tools.

33.) *In the fourth paragraph of page 16,*

*Previous:*

Moreover, there are limited number of other species with well-characterized and extensive complex lists in this database; thus, unlike homology mapping is an option, ProInfer may not work well on samples from other species (e.g., mouse, where PIA even reports more proteins from mouse RAW264.7 cell line data).

*Revised:*

Moreover, there are limited number of other species with well-characterized and extensive protein complex lists in this database; thus, unless homology mapping is an option, ProInfer may not work well on samples from other species (e.g., mouse, where PIA reports more proteins from mouse RAW264.7 cell line data).

34.) *In Conclusion at 17,*

*Previous:*

Critically, ProInfer can identify large numbers of validatable novel proteins not found by any other tool. In our evaluations, we find that these novel proteins are phenotype relevant. Thus, ProInfer is promising for functional profiling and discovering novel biomarkers or drug targets. Source codes of ProInfer are free accessible from <https://github.com/PennHui2016/ProInfer>.

*Revised:*

Critically, ProInfer can identify large numbers of validated novel proteins not found by any other tool. In our evaluations, we find that these novel proteins are phenotype relevant. Thus, ProInfer is promising for functional profiling and discovering novel biomarkers or drug targets. Source codes of ProInfer are publicly accessible at <https://github.com/PennHui2016/ProInfer>.

35.) *In Figure 3D, we put the wrong numbers of uniquely identified DEPs with literature proof or without literature proof for EPIFANY and Percolator. EPIFANY uniquely identified 0 DEP with literature proof and 1 DEP without literature proof while Percolator uniquely identified 1 DEP with literature proof and 0 DEP without literature proof*

*Previous:*

*Revised:*

36.) *We used 11806 proteins in Hela cell line and 11584 proteins in THP1 cell line (see* ***S4 Table****) that obtained from* *Human Protein Atlas (HPA) to validate proteins inferred by different protein inference tools. After removing proteins without protein level evidence from these two validation sets, there are 11692 and 11483 proteins in Hela and THP1 respectively. Using the two renewed validation sets instead, our results minorly changes in our results were obtained. We attached the comparison of results based on the old validation sets and new validation sets and the two new validation sets in* ***S7 Table****.*
37.) *In page 18, we revised the Autor Contributions,*

*Previous:*

HP developed ProInfer and wrote the manuscript. LW and WWBG supervised and co-wrote the manuscript.

*Revised:*

**Hui Peng**

ROLES: Conceptualization, Data Curation, Formal Analysis, Investigation, Methodology, Writing – Original Draft Preparation, Writing – Review & Editing.

AFFILIATION: Lee Kong Chian School of Medicine, Nanyang Technological University, Singapore; School of Biological Sciences, Nanyang Technological University, Singapore.

**Limsoon Wong**

ROLES: Supervision, Writing – Review & Editing

AFFILIATION: Department of Computer Science, National University of Singapore, Singapore.

**Wilson Wen Bin Goh**

ROLES: Supervision, Writing – Review & Editing

AFFILIATION: Lee Kong Chian School of Medicine, Nanyang Technological University, Singapore; School of Biological Sciences, Nanyang Technological University, Singapore; Center for Biomedical Informatics, Nanyang Technological University, Singapore.

38.) *In page 18,*

*Previous:*

**Wilson Wen Bin Goh** is an Assistant Professor of Biomedical Informatics, Director of Graduate Programme in Biomedical Data Science, Assistant Chair of Graduate Education at Lee Kong Chian School of Medicine, School of Biological Sciences, Nanyang Technological University, Singapore.

*Revised:*

**Wilson Wen Bin Goh** is Assistant Professor of Biomedical Informatics, Director of Graduate Programme in Biomedical Data Science, PhD Programme Director at Lee Kong Chian School of Medicine and School of Biological Sciences, Nanyang Technological University, Singapore.

39.) *In page 22, we added the supporting information, see below details,*

**Supporting information**

**S1 Table. Data for generating figures in the main text.** Sheets in S1_Table.xlsx with names “Figure 1C” and “Figure 1D” show the tables containing the data for generating **Figure 1C** and **Figure 1D** in our main text. Similarly, sheets “Figure 2A” to “Figure 2E” and “Figure 3A” to “Figure 3E” show the corresponding data for generating our **Figure 2A** to **Figure 2E** and **Figure 3A** to **Figure 3E** in main text respectively.

**S2 Table. Results of parameter optimization for competing protein inference tools.** The five Sheets with names “supp.tab1” to “supp.tab5” show the parameter optimization results for EPIFANY, Fido, Percolator, PIA and ProInfer.

**S3 Table. Literature proofs for validating uniquely found differentially expressed proteins based on different protein inference tools.** The seven sheets with names “supp.tab1” to “supp.tab7” show the literature proofs for validating uniquely found differentially expressed proteins based on five protein inference tools ProInfer, EPIFANY, Fido, Percolator, PIA and two quantification analysis platforms FragPipe and maxquant.

**S4 Table. Proteins obtained from the Human Protein Atlas for protein inference validation.** Sheets “Hela detectable Protein in HPA” and “THP1 detectable Protein in HPA” contain the lists of detectable proteins in Hela and THP1 from the Human Protein Atlas for validating protein inference performances.

**S5 Table. Comparison of methods “mean” and “max” for calculating protein complex confidence scores.** Sheet 1 shows the comparison results of using “mean” and “max” to calculate protein complex confidence scores.

**S6 Table. Results of testing the robustness of proposed ProInfer by removing pre-inferred proteins.** Sheet 1 shows the testing of robustness of ProInfer by removing 5~50% of pre-inferred proteins.

**S7 Table. The alternative validation data and the comparisons of validating inferred proteins with original validation data and the alternative validation data.** Sheets “HPA_Hela_protein_level” and “HPA_THP1_protein_level” give the two alternative validation data by removing proteins without protein level evidence. Sheet “parameter optimization” shows the detail parameter optimization results of different protein inference tools based on the alternative validation data in sheet “HPA_Hela_protein_level”. The Sheets “Figure 1C”, and “Figure 1D” show the minor changes in result data for generating our **Figure 1C** and **Figure 1D** in main text when using the alternative validation data in sheet “HPA_Hela_protein_level”. Sheets with names “Figure 2A” to “Figure 2E” show the minor changes when using the alternative validation data in sheet “HPA_THP1_protein_level” for performance tests of different protein inference tools.

Sincerely,

Hui Peng, Limsoon Wong and Wilson Goh
