# Supplementary material for: ProInfer: An interpretable protein inference tool leveraging on biological networks
Source: PLoS Comput Biol. 2023 Mar 17;19(3):e1010961. doi: 10.1371/journal.pcbi.1010961 (PMC10057851; doi:10.1371/journal.pcbi.1010961)
Supplement: Attachment — Submitted filename: response_letter.docx [file pcbi.1010961.s009.docx]

Dear Editor,

We would like to thank the reviewers and editorial again for their valuable inputs. We have revised our manuscript accordingly. The point-by-point responses are as follows:

**# Editor’s comments**

In particular you should carefully address the requested definition of your approach to defining false positive. Please respond carefully to the suggestions of the reviewer both in the first and second revision,

***Response:***

*We have added new tests to check whether common proteins, found across multiple complexes, may lead towards an inflation of false positives as suggested by reviewer2. The results are discussed as shown below.*

When you are ready to resubmit, please upload the following:

[1] A letter containing a detailed list of your responses to all review comments, and a description of the changes you have made in the manuscript. Please note while forming your response, if your article is accepted, you may have the opportunity to make the peer review history publicly available. The record will include editor decision letters (with reviews) and your responses to reviewer comments. If eligible, we will contact you to opt in or out.

***Response:***

*This is done.*

[2] Two versions of the revised manuscript: one with either highlights or tracked changes denoting where the text has been changed; the other a clean version (uploaded as the manuscript file).***Response:***

*This is done. We have submitted two versions of revised manuscript with the names of “main_text_with_revision_highlights.docx” and “main_text_with_revision_clean.docx” (please see attached submissions).*

**Comments of Review2**

**Reviewer #2:** I thank the authors for clarifying some of the points in their manuscript.

***Q1***. I do still have a concern about certain proteins found in many complexes. As mentioned before, some complexes may not be expressed even though a member is highly abundant (CALM1 as an example). This may lead the algorithm to improperly “enhance” the confidence score of a protein associated to a complex that is not expressed. This may be a source of false positives. In the authors’ response, they describe a scenario of two proteins P1 and P2 which have small confidence scores which map to two different complexes associated to CALM1, c1 and c2. P1 and P2 are both enhanced due to their association to c1 and c2. If we additionally know that c2 is not expressed in the tissue of study, P2’s confidence score was then erroneously enhanced. In the described algorithm, this scenario is only problematic with proteins that are found in many complexes such as CALM1.

A potential test would be to remove all proteins and their complexes from CORUM seen in > 3 complexes and rerun ProInfer. As a control, remove the same number of proteins/complexes that match the average abundances in the first set. This may show if the many complex proteins are having an effect.

***Response to Q1:***

*We checked the impact of removing proteins common to multiple complexes (multi-complex proteins) with tests as follows:*

***Step1****, we removed complexes and their proteins in CORUM if they contain at least one protein that shared by at least 4 complexes. There are 2371 out of 2916 such complexes. We then used the remaining 605 protein complexes as the input of ProInfer and tested it with Hela DDA1 data. We named this test as “Remove_3Overlap”.*

***Step2****, as control, we randomly removed 2371 complexes from CORUM (total 2916 complexes) and reran ProInfer on Hela DDA1 data. This process was repeated 10 times. We averaged the performances of these 10 times’ rerunning as the final performance. This test is named as “Remove_Random”. While 10 times may be seen as suboptimal, it is a computationally expensive process and we merely wanted to see if it produced similar outcomes as Step 1.*

***Step3****, we then checked the performance changes of “Remove_3Overlap” and “Remove_Random” against our original results (without complex removal, named as “ProInfer”).*

*Following figures show the performances of “Remove_3Overlap”, “Remove_Random” and “ProInfer”:*

**A**

Precision

|  | Remove_3Overlap | Remove_Random | ProInfer |
| --- | --- | --- | --- |
| 0.005_0.01 | 0.931402 | 0.931402 | 0.931402 |
| 0.005_0.05 | 0.976224 | 0.975425 | 0.972942 |
| 0.005_0.1 | 0.975234 | 0.974151 | 0.971195 |
| 0.005_0.25 | 0.975099 | 0.974233 | 0.971525 |
| 0.005_0.5 | 0.975383 | 0.975014 | 0.971685 |
| 0.005_0.75 | 0.972979 | 0.974491 | 0.967516 |
| 0.005_0.999 | 0.971592 | 0.974009 | 0.960906 |
| 0.01_0.01 | 0.931402 | 0.931402 | 0.931402 |
| 0.01_0.05 | 0.974005 | 0.973035 | 0.970714 |
| 0.01_0.1 | 0.973257 | 0.97202 | 0.969251 |
| 0.01_0.25 | 0.97301 | 0.971822 | 0.969454 |
| 0.01_0.5 | 0.973066 | 0.971574 | 0.969576 |
| 0.01_0.75 | 0.970034 | 0.970341 | 0.965008 |
| 0.01_0.999 | 0.968736 | 0.969613 | 0.960949 |
| 0.025_0.01 | 0.931402 | 0.931402 | 0.931402 |
| 0.025_0.05 | 0.916155 | 0.916155 | 0.916155 |
| 0.025_0.1 | 0.962906 | 0.961529 | 0.959223 |
| 0.025_0.25 | 0.962793 | 0.961693 | 0.960532 |
| 0.025_0.5 | 0.963795 | 0.962533 | 0.962401 |
| 0.025_0.75 | 0.961824 | 0.962124 | 0.960228 |
| 0.025_0.999 | 0.960458 | 0.961266 | 0.959062 |
| 0.05_0.01 | 0.931402 | 0.931402 | 0.931402 |
| 0.05_0.05 | 0.916155 | 0.916155 | 0.916155 |
| 0.05_0.1 | 0.908951 | 0.908951 | 0.908951 |
| 0.05_0.25 | 0.952502 | 0.951907 | 0.951072 |
| 0.05_0.5 | 0.952571 | 0.95167 | 0.952519 |
| 0.05_0.75 | 0.952301 | 0.953276 | 0.950349 |
| 0.05_0.999 | 0.952431 | 0.952199 | 0.949447 |

**B**

Recall

|  | Remove_3Overlap | Remove_Random | ProInfer |
| --- | --- | --- | --- |
| 0.005_0.01 | 0.363505 | 0.363505 | 0.363505 |
| 0.005_0.05 | 0.352456 | 0.354539 | 0.360615 |
| 0.005_0.1 | 0.354751 | 0.356799 | 0.36393 |
| 0.005_0.25 | 0.356111 | 0.358295 | 0.365375 |
| 0.005_0.5 | 0.356961 | 0.358134 | 0.3675 |
| 0.005_0.75 | 0.358066 | 0.356757 | 0.364525 |
| 0.005_0.999 | 0.360445 | 0.354666 | 0.382288 |
| 0.01_0.01 | 0.363505 | 0.363505 | 0.363505 |
| 0.01_0.05 | 0.35985 | 0.361584 | 0.366225 |
| 0.01_0.1 | 0.36189 | 0.363743 | 0.369709 |
| 0.01_0.25 | 0.36155 | 0.364057 | 0.369539 |
| 0.01_0.5 | 0.362315 | 0.364567 | 0.371069 |
| 0.01_0.75 | 0.363165 | 0.366165 | 0.377358 |
| 0.01_0.999 | 0.366055 | 0.369888 | 0.389002 |
| 0.025_0.01 | 0.363505 | 0.363505 | 0.363505 |
| 0.025_0.05 | 0.392827 | 0.392827 | 0.392827 |
| 0.025_0.1 | 0.372854 | 0.374282 | 0.377868 |
| 0.025_0.25 | 0.373874 | 0.375735 | 0.380588 |
| 0.025_0.5 | 0.375574 | 0.377503 | 0.382883 |
| 0.025_0.75 | 0.374724 | 0.377996 | 0.385773 |
| 0.025_0.999 | 0.377783 | 0.381871 | 0.396226 |
| 0.05_0.01 | 0.363505 | 0.363505 | 0.363505 |
| 0.05_0.05 | 0.392827 | 0.392827 | 0.392827 |
| 0.05_0.1 | 0.402176 | 0.402176 | 0.402176 |
| 0.05_0.25 | 0.383478 | 0.384889 | 0.388237 |
| 0.05_0.5 | 0.385773 | 0.38709 | 0.390447 |
| 0.05_0.75 | 0.385178 | 0.38737 | 0.393677 |
| 0.05_0.999 | 0.387982 | 0.391756 | 0.408635 |

**C**

F1 score

|  | Remove_3Overlap | Remove_Random | ProInfer |
| --- | --- | --- | --- |
| 0.005_0.01 | 0.522925 | 0.522925 | 0.522925 |
| 0.005_0.05 | 0.517922 | 0.520053 | 0.526198 |
| 0.005_0.1 | 0.520254 | 0.522298 | 0.529459 |
| 0.005_0.25 | 0.521696 | 0.52391 | 0.531036 |
| 0.005_0.5 | 0.522648 | 0.523848 | 0.5333 |
| 0.005_0.75 | 0.523484 | 0.522296 | 0.529539 |
| 0.005_0.999 | 0.52582 | 0.519974 | 0.546969 |
| 0.01_0.01 | 0.522925 | 0.522925 | 0.522925 |
| 0.01_0.05 | 0.525538 | 0.527243 | 0.531811 |
| 0.01_0.1 | 0.527601 | 0.529383 | 0.535253 |
| 0.01_0.25 | 0.527203 | 0.529687 | 0.535106 |
| 0.01_0.5 | 0.528024 | 0.530189 | 0.536726 |
| 0.01_0.75 | 0.528477 | 0.531691 | 0.542555 |
| 0.01_0.999 | 0.531335 | 0.535492 | 0.553815 |
| 0.025_0.01 | 0.522925 | 0.522925 | 0.522925 |
| 0.025_0.05 | 0.549878 | 0.549878 | 0.549878 |
| 0.025_0.1 | 0.537557 | 0.538823 | 0.542162 |
| 0.025_0.25 | 0.538598 | 0.540353 | 0.545167 |
| 0.025_0.5 | 0.540517 | 0.542312 | 0.54782 |
| 0.025_0.75 | 0.539327 | 0.542754 | 0.550415 |
| 0.025_0.999 | 0.542272 | 0.546596 | 0.560775 |
| 0.05_0.01 | 0.522925 | 0.522925 | 0.522925 |
| 0.05_0.05 | 0.549878 | 0.549878 | 0.549878 |
| 0.05_0.1 | 0.557624 | 0.557624 | 0.557624 |
| 0.05_0.25 | 0.54681 | 0.548144 | 0.551391 |
| 0.05_0.5 | 0.54915 | 0.550333 | 0.553861 |
| 0.05_0.75 | 0.548502 | 0.550884 | 0.556731 |
| 0.05_0.999 | 0.551362 | 0.555121 | 0.571361 |

*In the above figure, the x-axis shows the combination of protein reporting FDR and PSM filtering threshold, e.g., 0.005_0.01 means we inferred proteins by filtering out PSMs with PEP>0.01 and report proteins with FDR 0.005. The y-axis is the performance metric where panels A, B and C shows precision, recall and F1 score respectively. We can see, precisions of “Remove_3Overlap” and “Remove_Random” are quite similar to each other and are a bit higher than “ProInfer”. For Recall and F1, ProInfer works better. “Remove_Random” obtains slightly higher recall and F1 than “Remove_3Overlap”(differences are within 0.01). The small decrease of precision and increases of recall and F1 of ProInfer comparing to “Remove_3Overlap” and “Remove_Random” are mainly caused by the removal of a lot of complexes. The small differences of performances of “Remove_3Overlap” and “Remove_Random” suggests that the inclusion of multi-complex proteins, while a cause for concern, do not seem to have a strong adverse effect, especially when it comes to introducing false positives.*

*In the above tests, 2371 out of 2916 complexes were removed, thus the removed complex subsets of “Remove_3Overlap” and “Remove_Random” may overlap hugely. The small performance differences may be due to the small differences of complexes they finally used. In fact, not all the complexes are used by ProInfer. If we remove these “useless” complexes at first and then conduct above two kinds of tests again, less complexes may be removed. For example, when using PSM threshold of 0.01, 2281 complexes were used. In tests of “Remove_3Overlap” and “Remove_Random”, 972 complexes will be removed. Following figures show the results of our new tests.*

**D**

Precision

|  | Remove_3Overlap | Remove_Random | ProInfer |
| --- | --- | --- | --- |
| 0.005_0.01 | 0.931402 | 0.931402 | 0.931402 |
| 0.005_0.05 | 0.974122 | 0.973854 | 0.972942 |
| 0.005_0.1 | 0.972305 | 0.972184 | 0.971195 |
| 0.005_0.25 | 0.972456 | 0.972685 | 0.971525 |
| 0.005_0.5 | 0.972686 | 0.97321 | 0.971685 |
| 0.005_0.75 | 0.968618 | 0.971258 | 0.967516 |
| 0.005_0.999 | 0.962663 | 0.966379 | 0.960906 |
| 0.01_0.01 | 0.931402 | 0.931402 | 0.931402 |
| 0.01_0.05 | 0.971888 | 0.971544 | 0.970714 |
| 0.01_0.1 | 0.970364 | 0.970188 | 0.969251 |
| 0.01_0.25 | 0.970397 | 0.97049 | 0.969454 |
| 0.01_0.5 | 0.97116 | 0.970463 | 0.969576 |
| 0.01_0.75 | 0.966601 | 0.968037 | 0.965008 |
| 0.01_0.999 | 0.96226 | 0.964923 | 0.960949 |
| 0.025_0.01 | 0.931402 | 0.931402 | 0.931402 |
| 0.025_0.05 | 0.916155 | 0.916155 | 0.916155 |
| 0.025_0.1 | 0.959905 | 0.959872 | 0.959223 |
| 0.025_0.25 | 0.960379 | 0.960976 | 0.960532 |
| 0.025_0.5 | 0.963464 | 0.962562 | 0.962401 |
| 0.025_0.75 | 0.961588 | 0.962676 | 0.960228 |
| 0.025_0.999 | 0.959942 | 0.961566 | 0.959062 |
| 0.05_0.01 | 0.931402 | 0.931402 | 0.931402 |
| 0.05_0.05 | 0.916155 | 0.916155 | 0.916155 |
| 0.05_0.1 | 0.908951 | 0.908951 | 0.908951 |
| 0.05_0.25 | 0.951566 | 0.951623 | 0.951072 |
| 0.05_0.5 | 0.953271 | 0.953017 | 0.952519 |
| 0.05_0.75 | 0.951114 | 0.951885 | 0.950349 |
| 0.05_0.999 | 0.95056 | 0.951335 | 0.949447 |

**E**

Recall

|  | Remove_3Overlap | Remove_Random | ProInfer |
| --- | --- | --- | --- |
| 0.005_0.01 | 0.363505 | 0.363505 | 0.363505 |
| 0.005_0.05 | 0.358321 | 0.358338 | 0.360615 |
| 0.005_0.1 | 0.36104 | 0.360904 | 0.36393 |
| 0.005_0.25 | 0.36308 | 0.362885 | 0.365375 |
| 0.005_0.5 | 0.366225 | 0.363675 | 0.3675 |
| 0.005_0.75 | 0.369879 | 0.358703 | 0.364525 |
| 0.005_0.999 | 0.383478 | 0.369114 | 0.382288 |
| 0.01_0.01 | 0.363505 | 0.363505 | 0.363505 |
| 0.01_0.05 | 0.364355 | 0.364457 | 0.366225 |
| 0.01_0.1 | 0.36733 | 0.367304 | 0.369709 |
| 0.01_0.25 | 0.367755 | 0.36784 | 0.369539 |
| 0.01_0.5 | 0.369199 | 0.368859 | 0.371069 |
| 0.01_0.75 | 0.373874 | 0.372217 | 0.377358 |
| 0.01_0.999 | 0.387897 | 0.380112 | 0.389002 |
| 0.025_0.01 | 0.363505 | 0.363505 | 0.363505 |
| 0.025_0.05 | 0.392827 | 0.392827 | 0.392827 |
| 0.025_0.1 | 0.376424 | 0.376509 | 0.377868 |
| 0.025_0.25 | 0.379058 | 0.37882 | 0.380588 |
| 0.025_0.5 | 0.381013 | 0.380648 | 0.382883 |
| 0.025_0.75 | 0.382968 | 0.381421 | 0.385773 |
| 0.025_0.999 | 0.391042 | 0.3893 | 0.396226 |
| 0.05_0.01 | 0.363505 | 0.363505 | 0.363505 |
| 0.05_0.05 | 0.392827 | 0.392827 | 0.392827 |
| 0.05_0.1 | 0.402176 | 0.402176 | 0.402176 |
| 0.05_0.25 | 0.387387 | 0.38703 | 0.388237 |
| 0.05_0.5 | 0.390107 | 0.388926 | 0.390447 |
| 0.05_0.75 | 0.391892 | 0.390762 | 0.393677 |
| 0.05_0.999 | 0.403621 | 0.401895 | 0.408635 |

**F**

F1

|  | Remove_3Overlap | Remove_Random | ProInfer |
| --- | --- | --- | --- |
| 0.005_0.01 | 0.522925 | 0.522925 | 0.522925 |
| 0.005_0.05 | 0.523922 | 0.523901 | 0.526198 |
| 0.005_0.1 | 0.526557 | 0.526395 | 0.529459 |
| 0.005_0.25 | 0.528746 | 0.528572 | 0.531036 |
| 0.005_0.5 | 0.532107 | 0.529487 | 0.5333 |
| 0.005_0.75 | 0.535334 | 0.523908 | 0.529539 |
| 0.005_0.999 | 0.548471 | 0.53419 | 0.546969 |
| 0.01_0.01 | 0.522925 | 0.522925 | 0.522925 |
| 0.01_0.05 | 0.530012 | 0.530068 | 0.531811 |
| 0.01_0.1 | 0.532922 | 0.532869 | 0.535253 |
| 0.01_0.25 | 0.533374 | 0.533477 | 0.535106 |
| 0.01_0.5 | 0.535008 | 0.534544 | 0.536726 |
| 0.01_0.75 | 0.539192 | 0.537688 | 0.542555 |
| 0.01_0.999 | 0.552911 | 0.545378 | 0.553815 |
| 0.025_0.01 | 0.522925 | 0.522925 | 0.522925 |
| 0.025_0.05 | 0.549878 | 0.549878 | 0.549878 |
| 0.025_0.1 | 0.540781 | 0.540864 | 0.542162 |
| 0.025_0.25 | 0.543571 | 0.543422 | 0.545167 |
| 0.025_0.5 | 0.546075 | 0.545554 | 0.54782 |
| 0.025_0.75 | 0.547775 | 0.546366 | 0.550415 |
| 0.025_0.999 | 0.55571 | 0.554201 | 0.560775 |
| 0.05_0.01 | 0.522925 | 0.522925 | 0.522925 |
| 0.05_0.05 | 0.549878 | 0.549878 | 0.549878 |
| 0.05_0.1 | 0.557624 | 0.557624 | 0.557624 |
| 0.05_0.25 | 0.550616 | 0.550265 | 0.551391 |
| 0.05_0.5 | 0.553646 | 0.552412 | 0.553861 |
| 0.05_0.75 | 0.555074 | 0.554069 | 0.556731 |
| 0.05_0.999 | 0.566639 | 0.565072 | 0.571361 |

Again, across panels D to F, we don’t see serious affections of multi-complex proteins on ProInfer’s performance. In conclusion, inclusion of multi-complex proteins rarely introduces more false positives.

***Q2:*** The use of the terms “validated” and “non-validated” is confusing as it suggests using additional experimental evidence for validation. Using terms “true-positives” and “false-positives” seems sufficient.

***Response to Q2:***

*We replaced “validated” with “true-positives” and replaced “non-validated” with “false-positives” in our manuscript. We highlighted these modifications in file main_text_with_revision_highlights.docx in red. Following are tracks of our modifications, we underline those original contents that have been modified in revised version and color corresponding revised contents in red (page numbers are counted in main_text_with_revision_highlights.docx). Below are the detail modifications:*

1.) *In Abstract at page 3,*

*Previous:*

ProInfer always infers the most numbers of validated proteins, in comparison to mainstream protein inference tools Fido, EPIFANY and PIA.

*Revised:*

ProInfer always infers the most numbers of true positives, in comparison to mainstream protein inference tools Fido, EPIFANY and PIA.

2.) *In first paragraph of section “**Validation and performance evaluation” at page 10,*

*Previous:*

We label the proteins predicted by different protein inference tools, e.g., our ProInfer, EPIFANY, etc., and validated by the Human Protein Atlas as “Validated” (validated proteins are true positives), otherwise they are considered “Non-validated” (false positives). We calculate several metrics for evaluating competing tools and optimizing their hyperparameters including inferred protein numbers, validated protein numbers, recall, precision and F1 score. Recall, precision and F1 score are given by

*Revised:*

We label the proteins predicted by different protein inference tools, e.g., our ProInfer, EPIFANY, etc., and validated by the Human Protein Atlas as true positives, otherwise they are considered false positives. We calculate several metrics for evaluating competing tools and optimizing their hyperparameters including inferred protein numbers, numbers of true positives, recall, precision and F1 score. Recall, precision and F1 score are given by

3.) *In the caption of Figure 1 and the legend of Figure 1C at page 11,*

*Previous:*

**Figure 1** **Workflow for protein inference and differential expression analysis with proteomic data and hyperparameter optimization results of proposed ProInfer.** A shows a simple workflow of proteomics data-based protein inference and differential expression analysis. B gives a schema diagram of how ProInfer works. C presents the average inferred protein numbers and validated numbers by Human Protein Atlas (HPA) achieved by ProInfer under different psm filtering thresholds (y-axis). The curves in D illustrate the performances of ProInfer indicated by recall, F1 (left-y axis) and precision (right-y axis) affected by various psm filtering thresholds (x-axis).

*Revised:*

**Figure 1** **Workflow for protein inference and differential expression analysis with proteomic data and hyperparameter optimization results of proposed ProInfer.** A shows a simple workflow of proteomics data-based protein inference and differential expression analysis. B gives a schema diagram of how ProInfer works. C presents the average inferred protein numbers and numbers of true positives found by ProInfer under different psm filtering thresholds (y-axis). The curves in D illustrate the performances of ProInfer indicated by recall, F1 (left-y axis) and precision (right-y axis) affected by various psm filtering thresholds (x-axis).

4.) *In the last paragraph of page 12,*

*Previous:*

In **Figure 2A**, we showed the proportions of validated proteins (deep colors) against non-validated proteins (light colors). Protein reporting FDRs were set as 0.005, 0.01, 0.025 and 0.05 respectively. Regardless of FDR threshold, ProInfer reports the most numbers of validated proteins (albeit, with correspondingly more non-validated ones as well). For instance, given FDR 0.01, ProInfer reports 5390 proteins in total, of which, 5041 are validated proteins. Compared against Percolator, 640 (**Figure 2A**, 5041-4401) more validated proteins were reported with just 230 (**Figure 2A**, 349-119) more non-validated ones, achieving a 1:2.78 ratio of non-validated:validated gain (230:640). Similarly, the non-validated:validated gains comparing to EPIFANY, Fido and PIA are 1:2.90, 1:6.1 and 1:9.37. In addition, from **Figure 2A**, even with looser FDR thresholds, e.g., 0.025 and 0.05, ProInfer reports more validated proteins without incurring great changes to the presence of non-validated proteins (about 10 more non-validated comparing to FDR 0.005 or 0.01). From **Figure** **2B**, ProInfer produces stable precisions while other tools acquire lower precisions as FDRs relaxes. In **Figure 2 (C** & **D)**, the line plots show that ProInfer always achieves the highest recall and F1 score. Notably, all methods obtain better recalls and F1 scores when FDR relaxes from 0.005 to 0.05. Amongst the methods, Percolator always gets highest precisions but lowest recalls and F1 scores.

*Revised:*

In **Figure 2A**, we showed the proportions of true positives (deep colors) against false positives (light colors). Protein reporting FDRs were set as 0.005, 0.01, 0.025 and 0.05 respectively. Regardless of FDR threshold, ProInfer reports the most numbers of true positives (albeit, with correspondingly more false positives as well). For instance, given FDR 0.01, ProInfer reports 5390 proteins in total, of which, 5041 are true positives. Compared against Percolator, 640 (**Figure 2A**, 5041-4401) more true positives were reported with just 230 (**Figure 2A**, 349-119) more false positives, achieving a 1:2.78 ratio of false positives:true positives gain (230:640). Similarly, the false positives:true positives gains comparing to EPIFANY, Fido and PIA are 1:2.90, 1:6.1 and 1:9.37. In addition, from **Figure 2A**, even with looser FDR thresholds, e.g., 0.025 and 0.05, ProInfer reports more true positives without incurring great changes to the presence of false positives (about 10 more false positives comparing to FDR 0.005 or 0.01). From **Figure** **2B**, ProInfer produces stable precisions while other tools acquire lower precisions as FDRs relaxes. In **Figure 2 (C** & **D)**, the line plots show that ProInfer always achieves the highest recall and F1 score. Notably, all methods obtain better recalls and F1 scores when FDR relaxes from 0.005 to 0.05. Amongst the methods, Percolator always gets highest precisions but lowest recalls and F1 scores.

5.) *In legend of Figure 2A and caption of Figure 2 at page 14,*

*Previous:*

**Figure 2. Performance evaluation of various tools tested on 3 replicates of human THP1 dataset. A** shows average numbers of validated (in deep colors) and non-validated (in light colors) target proteins reported in 3 replicates of THP1 sample with FDR of 0.005, 0.01, 0.025 and 0.05 respectively with hyperparameters optimized with Hela data. **B, C & D** show the changes of precision, recall and F1 values of competing tools under different protein reporting FDRs. **E** is a Venn diagram revealing the overlap of proteins reported by different tools and validation status of proteins that reported only by a specific tool.

*Revised:*

**Figure 2. Performance evaluation of various tools tested on 3 replicates of human THP1 dataset. A** shows average numbers of true positives (in deep colors) and false positives (in light colors) reported in 3 replicates of THP1 sample with FDR of 0.005, 0.01, 0.025 and 0.05 respectively with hyperparameters optimized with Hela data. **B, C & D** show the changes of precision, recall and F1 values of competing tools under different protein reporting FDRs. **E** is a Venn diagram revealing the overlap of proteins reported by different tools and validation status of proteins that reported only by a specific tool.

6.) *In the first paragraph of Discussion at page 15,*

*Previous:*

Most protein inference methods except ProInfer, identified fewer than 5000 validated proteins even at a loose protein FDR of 0.05 (**Figure 2A**).

*Revised:*

Most protein inference methods except ProInfer, identified fewer than 5000 true positives even at a loose protein FDR of 0.05 (**Figure 2A**).

***Additional revisions***

*We corrected a grammar error in our manuscript.*

*1). In section* ***“*While ProInfer dominates in our benchmarks, it does have drawbacks”** *of Discussion at page 16,*

*Previous:*

A strict filtering criterion may stave off more decoy peptides, which benefits some tools e.g., EPIFANY and Fido (see **Result**).

*Revised:*

A strict filtering criterion may stave off more decoy peptides, which benefits some tools e.g., EPIFANY and Fido (see **Results**).

*2). In Acknowledgements at page 17 & 18,*

*Previous:*

WWBG and LW acknowledge support from an MOE AcRF Tier 2 award (MOE2019-T2-1-042).

WWBG acknowledges support from an MOE AcRF Tier 1 award (RT11/21).

*Revised:*

Wilson Wen Bin Goh and Limsoon Wong acknowledge support from an MOE AcRF Tier 2 award (MOE2019-T2-1-042).

Wilson Wen Bin Goh acknowledges support from an MOE AcRF Tier 1 award (RT11/21).

Sincerely,

Hui Peng, Limsoon Wong and Wilson Goh
